# A Systematic Review of Cognitive Predictors of Treatment Outcome in Major Depression

**DOI:** 10.3389/fpsyt.2018.00382

**Published:** 2018-08-28

**Authors:** Samantha J. Groves, Katie M. Douglas, Richard J. Porter

**Affiliations:** ^1^Department of Psychological Medicine, University of Otago, Christchurch, New Zealand; ^2^Specialist Mental Health Services, Canterbury District Health Board, Christchurch, New Zealand

**Keywords:** major depression, cognitive predictors, cognitive function, treatment response, relapse, remission, executive function

## Abstract

**Background:** Research suggests that only 50% of patients with major depression respond to psychotherapy or pharmacological treatment, and relapse is common. Therefore, there is interest in elucidating factors that help predict clinical response. Cognitive impairment is a key feature of depression, which often persists beyond remission; thus, the aim of this systematic review was to determine whether baseline cognitive functioning can predict treatment outcomes in individuals with depression.

**Method:** Studies examining cognitive predictors of treatment response in depression were identified using Pub Med and Web of Science databases. Given the heterogeneity of outcome measures, the variety of treatment protocols, and the differing ways in which data was presented and analyzed, a narrative rather than meta-analytic review technique was used.

**Results:** 39 studies met inclusion criteria. Findings in younger adult samples were inconclusive. There was some evidence for a predictive effect of executive function and to a lesser extent, psychomotor speed, on treatment response. There was no evidence of learning or memory being associated with treatment response. In older-aged samples, the evidence was much more consistent, suggesting that poor executive function predicts poor response to SSRIs.

**Conclusions:** Findings from the present review suggest that certain aspects of cognitive functioning, particularly executive function, may be useful in predicting treatment response in depression. This is certainly the case in elderly samples, with evidence suggesting that poor executive functioning predicts poor response to SSRIs. With further research, baseline cognitive functioning may serve as a factor which helps guide clinical decision making. Moreover, cognitive deficits may become targets for specific pharmacological or psychological treatments, with the hope of improving overall outcome.

## Introduction

Major depression is among the leading causes of global disability (1) and although our understanding of the disorder is growing, treatment outcomes remain unsatisfactory. Research indicates that only 50% of patients respond to psychotherapy or pharmacological treatment and relapse is common (2, 3). Clinical factors predict differential response to treatments to a limited extent, leaving clinicians to choose first-line treatment on the basis of likely side effects, availability and their own clinical experience (4). With each treatment failure, there is an increased risk of both longer-term failure to respond to treatment and of relapse (5). In this context, there has been increased interest in elucidating factors that help predict clinical response in depression, including cognitive factors, hormonal measures, and neural markers.

Cognitive functioning is relatively easy to measure in clinical practice, and if found to be predictive of treatment response, it has the potential to be widely used. Evidence indicates that depression is associated with widespread cognitive deficit, including impairments in executive functioning, attention, verbal learning and memory, visual learning and memory, emotional processing and psychomotor speed (6). Although aspects of these cognitive deficits may resolve following successful treatment for some individuals, it is often the case that they persist beyond remission (7, 8).

If baseline cognitive deficits are predictive of eventual response, then such deficits could be targeted by specific pharmacological or psychological treatments, in the hope of improving overall outcome. For example, the antidepressant Vortioxetine has been shown to improve psychomotor and verbal memory function in moderate to severe depression (9), while RU486 has been shown to improve spatial working memory in the depressed phase of bipolar disorder (10). Considerable research is currently occurring into psychological techniques that aim to improve cognitive function in depression (11). Indeed, studies that have specifically targeted executive dysfunction in elderly depressed patients, have found positive effects (12). However, due to the intensive nature of such psychological treatments, it is likely that these techniques need to be aimed at those who would have otherwise experienced a more difficult and prolonged recovery. A further implication of finding cognitive predictors of treatment response is that if cognitive impairment is known to predict poorer outcomes, then this may prompt a more aggressive approach in the initial stages of treatment. For example, a clinician may use a combination of psychotherapy and pharmacotherapy in situations where only one of these modalities would have been typically used.

The aims of the present review were therefore as follows: (i) to examine findings from studies investigating cognitive predictors of treatment response in depression, and (ii) to examine the methodological issues arising from the studies that have examined this. We reviewed all the available literature in which cognitive testing was conducted at baseline, to determine whether aspects of cognitive functioning would impact on treatment outcomes.

### Research questions

1. Does baseline cognitive functioning predict treatment outcomes in major depression?
2. Is the predictive relationship dependent on treatment modality?

## Methods

### Protocol and registration

Details of the protocol for this systematic review were registered on PROSPERO (42018081980) and can be accessed at www.crd.york.ac.uk/PROSPERO/display_record.asp?ID=CRD42018081980.

### Search strategy

Up to 1 December 2017, a systematic review of electronic databases was carried out for relevant papers using Pub Med and Web of Science. In the initial search, the following search items were used “major depression” or “depression” and “neuropsychological predictors” or “cognitive predictors” and “treatment response.” To ensure inclusion of all available articles, reference lists of all relevant papers were checked. Further, Web of Science was used to review articles that had cited the relevant articles found using the aforementioned search strategies, enabling the inclusion of more recent publications.

### Inclusion criteria

Any peer-reviewed article involving baseline assessment of cognitive functioning, a proposed active treatment of depression and a follow up measure of depression severity, were included in the present review. All subtypes of depression were also included (unipolar or bipolar - depressed phase, psychotic or non-psychotic). “Treatment” could be pharmacotherapy, electroconvulsive therapy (ECT), transcranial stimulation (direct current or magnetic), psychotherapy, or cognitive remediation (CR). Studies were required to use adult samples, with all participants 18 years of age or older.

### Exclusion criteria

Reasons for exclusion were: (i) use of a depressed sample with comorbid major medical, neurological or endocrinological conditions, (ii) inclusion of individuals scoring < 24 on a Mini Mental Status Exam (*n* = 6), and (iii) not presenting data on baseline depression severity (*n* = 1). All studies were limited to English-language publications.

### Full study review

Articles were initially screened by two of the reviewers who independently reviewed the titles and abstracts of studies, to accept or reject for full text review. The same two reviewers then examined the full texts of the studies that had passed initial screening, to determine if they still met inclusion criteria. If inclusion of a paper was unclear, then all three co-authors discussed in order to achieve a consensus. Data was extracted from eligible studies into a spreadsheet. For each study, we extracted the following data: (1) characteristics of the sample, including sample size, average age and baseline depression severity, (2) study design, (3) cognitive tests used during assessment, (4) response/remission criteria, and (5) study outcomes.

## Results

### Study characteristics

**Figure 1:**
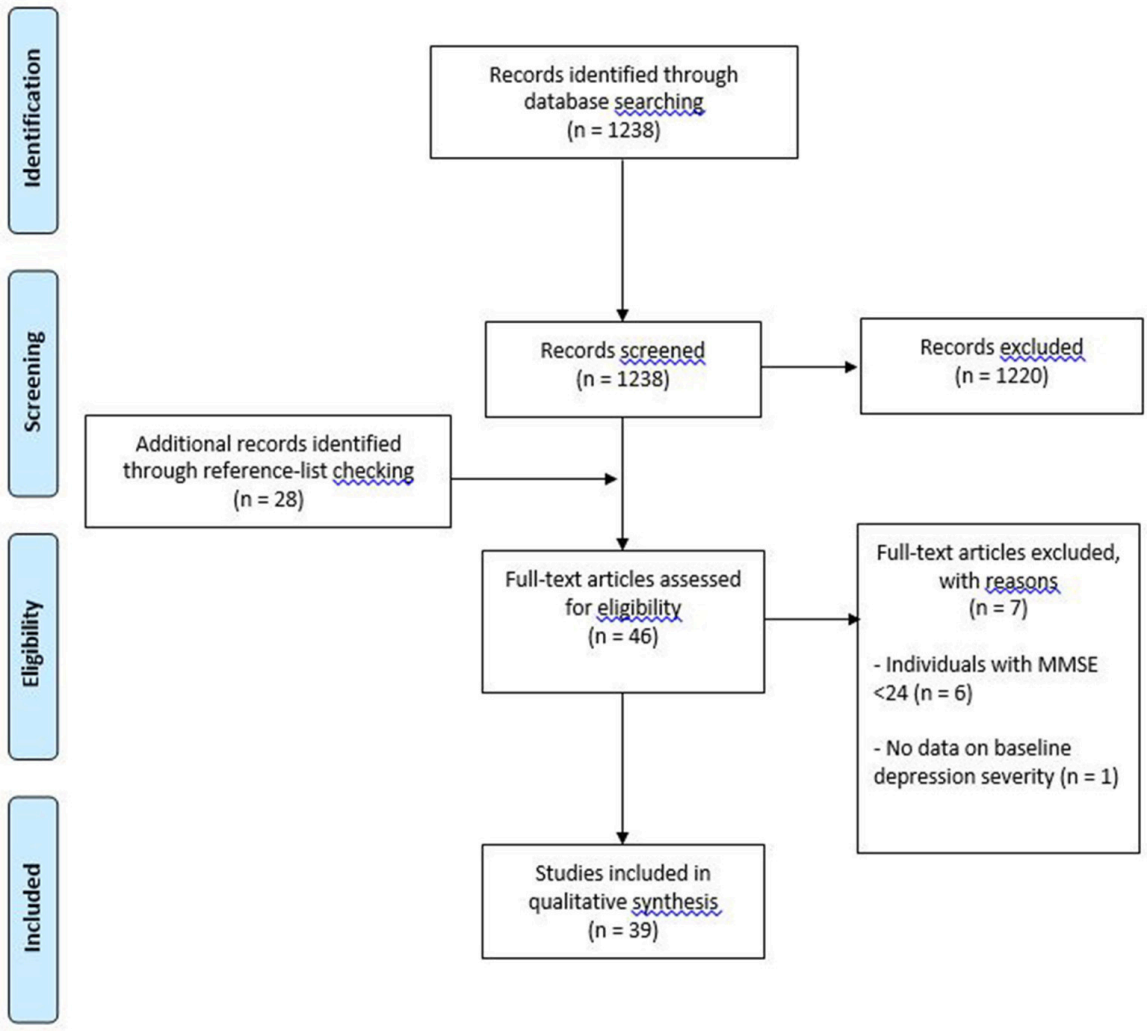
PRISMA flow diagram of studies retrieved for the review.

Thirty-nine studies met inclusion criteria (see Figure 1 for flow diagram of studies retrieved for review). Of these studies, 32 used pharmacotherapy as the primary treatment (18 single antidepressant, 14 mixed antidepressant treatment); 1 study used pharmacotherapy, psychotherapy and a combination thereof; 1 study used unrestricted pharmacotherapy treatment in addition to ECT (the latter being the final treatment option); 1 study used transcranial direct current stimulation; 1 study used deep brain stimulation, and 3 studies used psychosocial interventions (see Tables 1, 2). Regarding the term “mixed antidepressant treatment,” we refer to a number of possible situations. Firstly, open label treatment with a specific type of antidepressant but with the option to use various antidepressants within that class; secondly, open label treatment with any type of antidepressant; and thirdly, a standardized treatment algorithm allowing for treatment changes according to response. In one of the pharmacological studies (47), a small proportion of the sample received ECT in conjunction with pharmacotherapy (4 out of 100 participants). Given the small number of participants receiving adjunctive ECT, it was decided to group this study with others involving mixed antidepressant treatment. An additional pharmacological study (55) utilized ECT as a final treatment option. Given that 48% of the participants were treated with ECT, it was decided to group this study with the “other biological” treatment studies. One study utilized a naturalistic treatment protocol (38), which meant that some of the participants received no recognized treatment (*n* = 4). Because most participants received some form of antidepressant medication, it was decided to include the study in this review.

**Table 1:** Reviewed studies using antidepressant treatment during the follow-up period.

**Study**	**N**	**Age (SD)**	**Depression severity**	**Study design**	**Cognitive tests**	**Response/remission criteria**	**Key outcomes**
**ANTIDEPRESSANT MONOTHERAPY**							
(13)	14 MDE	41.86 (13.77)	HDRS: 22.86	9-week randomized, double-blind placebo controlled trial of Fluoxetine.	WAIS-R Digit Span, Digit Symbol & Block Design; TMT-A & B; Stroop parts A, B & C, Boston Naming Test, Rey Complex Figure Test, Benton Faces, RAVLT, WMS-R Visual Reproduction; WCST; Auditory Consonant Trigrams; COWAT.	Responder = HDRS <10 and no longer met criteria for MDE	8 responders, 6 non-responders to active treatment. Non-responders significantly worse performance on WCST and more errors on Stroop Test.
(14)	37 MDD	Responders – 37.92 (10.77) Non-responders-33.08 (9.38)	HDRS17: Responders-16.16 (3.57) Non-responders-17.33 (5.74)	12-week open trial of Fluoxetine	COWAT; SCWT; WCST; WAIS-III Digit Symbol, Block Design, Digit Span &Vocabulary.	Responders-no longer met criteria for MDD and had CGI scale score of much improved or very much improved.	25 responders, 12 non-responders. Non-responders had fewer words on COWAT and named fewer colors on Stroop (ES of 1.44 and 0.74 respectively). COWAT scores significantly predicted outcome of HDRS scores.
(15)	26 MDD outpatients	24.46 (4.72)	HDRS-17: 24.75 (5.61)	8-week trial of Bupropion-SR (150 mg/d)	WAIS III Digit Span, Delayed Match to Sample, Spatial Span, RAVLT, Pattern Recognition Memory, Paired Associates Learning, Spatial Recognition Memory, Match to Sample Visual Search, Reaction Time, RVIP, Stroop, COWAT, Intra-Extra Dimensional Set Shift, Spatial Working Memory, Stockings of Cambridge.	Response ≥ 50% decrease in HDRS-17 at end of 8 weeks.	12 responders, 8 non-responders. Responders performed more poorly on Paired Associates Learning and Stockings of Cambridge at baseline (small ES).
(16–18)	72 MDD	31.18 (7.56)	HDRS-17: 21.47 (2.81)	12-week Fluoxetine treatment (20 mg/d)	WAIS III Digit Span, Spatial Working Memory, RAVLT, Paired Associates Learning, Delayed Matching to Sample, Stroop, RVIP, COWAT, Intra-Extra Dimensional Set Shift, Stockings of Cambridge.	Response ≥ 50% decrease in HDRS-17 at end of 4 weeks. Remission = HDRS-17 of 6 or lower at 12 weeks.	2010: After 4 weeks, 42 responders, 22 non-responders, 8 dropouts. Responders performed better on Digit Span forwards and were faster with their initial thinking time on Stockings of Cambridge compared with non-responders. In contrast, responders had slower mean subsequent thinking time on Stockings of Cambridge. 2012: Analyses included data from 56 participants. Forty-three participants remitted by 12 weeks; however, no cognitive variables predicted remission. 2013: Analyses included all 72 participants. Of the 51 remitters, those with slower processing speed (Stroop) and poorer Spatial Working Memory performance were slower to remit.
(19)	13 MDD (treatment resistant)	52	HDRS-17: 20.3 (4.3)	Six infusions of Ketamine Hydrochloride (0.5 mg/kg over 40 min) over 12-day period	CogState Battery	Response = ≥ 50% reduction in MADRS score. Remission = ≤ 9 MADRS.	The likelihood of responding to six Ketamine infusions was greater amongst those with poor attention at baseline.
(20)	1008 MDD (655 completers) 336 HC	MDD: Intact - 35 (11.6) Impaired-45.6 (11.9) HC - 37.0 (13.1)	HDRS-17: Intact- 21.7 (3.9) Impaired- 22.4 (4.5)	8-week trial of either Escitalopram, Sertraline or Venlafaxine-ER.	Motor Tapping; Choice Reaction Time; Memory Recall; Digit Span; SCWT; Continuous Performance Test; Go/No-Go, Switching of Attention; Executive Maze; Explicit Emotion Identification & Emotion Attention Bias.	Remission = HDRS-17 ≤ 7 or QIDS ≤ 5. Response ≥ 50% decrease on HDRS or QIDS	Cluster analysis showed MDD participants fell into 2 sub-groups: intact (735) and impaired (273); the latter, performed below the healthy norm for 11/13 aspects of functioning. Impairments greatest in patients predicted to be non-remitters to Escitalopram for attention, decision speed, working memory and speed of emotion identification.
(21)	25 MDD	43.7 (12.5)	HDRS-17 = 22.2 (4.9)	6-week Duloxetine treatment (65.8 mg ± 16.1)	Test Battery for Attentional Performance.	Response = ≥ 50% reduction in HDRS score	Greater alertness and divided attention were associated with lower HDRS scores, post-treatment.
(22)	25 MDD (treatment resistant)	Responders: 53.81 Non-responders: 40.44	MADRS: Responders = 36.88 Non-responders = 37	Open label, single infusion of Ketamine Hydrochloride (0.5 mg/kg over 40 min)	Tests from MCCB: TMT-A, WMS III Spatial Span, BACS Digit Symbol, Letter-Number Sequencing, Hopkins Verbal Learning Test, Brief Visual Memory Test, Category Fluency, Continuous Performance Test.	Response = ≥ 50% reduction in MADRS score, 24 h following baseline assessment.	Psychomotor speed predicted response to Ketamine. Responders were significantly more impaired than non-responders in the domains of psychomotor speed, working memory and composite MCCB.
(23)	43 MDD (treatment resistant)	47.1 (12.6)	MADRS = 32.5 (6.0)	Double-blind, single infusion of either Ketamine Hydrochloride (0.5 mg/kg) or Midazolam (0.045 mg/kg) over 40 min; latter served as active placebo.	Tests from MCCB: TMT-A, WMS III Spatial Span, BACS Digit Symbol, Letter-Number Sequencing, Hopkins Verbal Learning Test, Brief Visual Memory Test, Neuropsychological Assessment Battery Mazes, Category Fluency.	Response = ≥ 50% reduction in MADRS score. Primary outcome was change in MADRS score 24 h following treatment. Secondary outcomes included MADRS score at 48 and 72 h and 7 days following treatment.	Psychomotor speed predicted response to Ketamine, whereby slow psychomotor speed at baseline was associated with improved antidepressant response to Ketamine. Responders were significantly more impaired than non-responders in the domain of psychomotor speed.
(24)	508 MDD	Remitters −47 (12.7) Non-remitters−45.2 (11.9)	QIDS-SR: Remitted-15.5 (5.2) NR-16.1 (4.4)	Multicentre study conducted in naturalistic setting in 388 community psychiatric centers. 6–8 weeks of Agomelatine (25–50 mg)	D2 Cancellation Test; TMT-A & B.	QIDS-SR ≤ 5 at 6–8 weeks	The number of omission mistakes on D2 (attention) predicted clinical and functional remission (with a dose-effect). Fewer mistakes associated with better outcomes.
(25)	272 MDD	Long term psychodynamic psychotherapy-−29.9 (2.43) Fluoxetine-29.64 (2.21) Combination-29.39 (1.01)	BDI: Long term psychodynamic psychotherapy−27.36 (3.82) Fluoxetine-29.64 (2.71) Combination-29.39 (3.85)	24 months of long-term psychodynamic psychotherapy (*n* = 90), Fluoxetine treatment (*n* = 91) or a combination thereof (*n* = 90)	WAIS III: Vocabulary, Similarities, Arithmetic, Digit Span, Information, Comprehension, Letter-number Sequencing, Picture Completion, Digit-symbol Coding, Block Design, Matrix Reasoning, Picture Arrangement, Symbol Search, Object Assembly.	Conducted mixed model analyses-no remission/response criteria	Higher Letter-number Sequencing and Matrix Reasoning scores at baseline, predicted lower BDI scores at 24 months. Higher Similarities scores at baseline predicted higher BDI scores at 24 months. Higher baseline Digit-Symbol Coding scores predicted lower BDI scores in patients who received Fluoxetine, and higher BDI scores in patients receiving psychodynamic therapy or combined treatment, at 24 months.
(26)	22 MDE	Remitted-70.2 (7.4). Non-remitters-74.9 (8.1)	HDRS: Remitters-21.7. Unremitters-25.4	6-week trial of Citalopram	MDRS-five domains: attention, Initiation/Perseveration, construction, conceptualisation and memory.	Remission ≤ 10 HDRS	13 patients remitted and 9 remained symptomatic. I/P scores were lower in those who did not remit vs. those who did.
(27)	444 MDD (217 Sertraline, 119 Fluoxetine, 104 Nortriptyline)	Sertraline group - 68.0 (5.7) Fluoxetine group - 67.4 (5.9) Nortriptyline group - 67.9 (6.6)	HDRS-24: Sertraline group-24.9 (4.6) Fluoxetine group-25.0 (4.7) Nortriptyline group-24.8 (5.2)	Two double-blind 12-week studies comparing Sertraline (50 mg per day), to Fluoxetine (20 mg per day) and to Nortriptyline (25 mg per day).	Buschke-Fuld Selective Reminding Test, Digit Symbol Substitution Task & MMSE.	Responder status defined as a CGI-I score of “much” or “very much” improved.	Cognitive scores did not significantly predict endpoint improvement in depression, nor time to respond.
(28, 29)	112 MDD	Remitters-71.56 (6.4) Non-remitters-75.1 (6.15)	HDRS: Remitters-23.13 Non-remitters-25.76	8-week trial of Citalopram	Initiation/Perseveration subscale of the MDRS and SCWT	Remission ≤ 10 HDRS Responders ≥ 50% change on HDRS.	2004: 61 remitters and 51 non-remitters. Lower I/P scores were associated with longer time to remission and poor remission rate. Lower Stroop scores were associated with poor remission rate. 2005: I/P scores below the median ( ≤ 35) and Stroop scores at the lowest quartile (≤ 22) predicted less change in depressive symptoms. Thus, poorer executive function performance was associated with poor treatment response.
(30)	12 MDD	Remitters −71.2 (5.0) Non-remitters-68.8 (6.3)	HDRS: Remitters-19.2 (2.6) Non-remitters-22.0 (6.7)	8-week open-controlled trial of Escitalopram (10 mg/day).	MMSE, MDRS, Hopkins Verbal Learning Test, WCST, Emotional Go/No-Go	Student's *t*-test between remitters and non-remitters	No significant differences between remitters and non-remitters in overall cognitive impairment, memory, performance on the WCST, and error rates or reaction time on the Emotional Go/No-Go Task.
(31)	13 MDD 13 HC	71.5 (6.7)	HDRS-24: 18.	8-week trial of Citalopram	Attention Network Test	Time to remission: 1st day of a 2-week period where a patient didn't meet diagnostic criteria and HDRS < 10.	Significant correlation between conflict scores (measure of executive function) and time to remission. Those with greater cost reaction time due to incongruent flankers took longer to remit.
(32, 33)	84 MDD	79	HDRS-24: 24	8-week multisite, randomized, placebo controlled trial of Citalopram (20–40 mg/d)	2007: SCWT measured response inhibition (defines as the highest quartile of the distribution). 2008: MMSE, WASI-III Digit Symbol Substitution, Choice Reaction Time, Judgement of Line Orientation, Buschke Selective Reminding Test.	2007: Using growth curves to examine the association between baseline response inhibition and depression severity at 8 weeks. 2008: Remission = HDRS < 10 Response = 50% reduction in HDRS over 8-week treatment period.	2007: Individuals classified as having high response inhibition had higher HDRS scores (poorer response) at 8 weeks, than those who did not. 2008: No association between treatment response and cognitive impairment. Impairment on Digit Symbol test was associated with slower treatment response.
(34)	70 MDD	Remitters: 70.1 (5.8) Non-remitters: 70.4 (7.1)	HDRS-24: Remitters = 21.8 (4.1) Non-remitters = 22.4 (3.7)	12-week Escitalopram trial (10 mg/d).	MDRS Initiation/Perseveration subscale, Simple Verbal Initiation/Perseveration, TMT-A, Hopkins Verbal Learning Test-Revised, WCST.	Remission = HDRS-24 ≤ 7 for 2 consecutive weeks and no longer met DSM-IV criteria for depression.	Worse performance on MDRS I/P (particularly complex verbal subscale) associated with poorer remission rates.
(35)	53 MDD 30 HC	MDD-72.18 (7.56) HC-72.83 (5.95)	HDRS: 23.4 (3.9)	12-week Escitalopram (target daily dose 20 mg).	SCWT, Tower of London, MDRS- Initiation/Perseveration and Iowa Gambling Test.	Remission-HDRS ≤ 10.	Individuals who were impaired on the Stroop, Tower of London and MDRS- I/P had a smaller reduction in depressive symptoms than those who were only impaired on the Iowa Gambling Task or those who were unimpaired. Further, this group demonstrated a lower probability of achieving remission than the other groups.
**MIXED ANTIDEPRESSANT TREATMENT**							
(36)	16 single MDE 32 recurrent MDE 4 BD	Responders−48.73 (11.04) Non-responders-49.28 (10.14)	HDRS-17: Responders-48.73 (11.04) Non-responders-49.28 (10.14)	First phase, Maprotiline or Nortryptiline and co-medication with Lunitrazepam, Lormetazepam or placebo. Second phase, non-responders switched from trycyclics, to either Brofaromine or Tranylcypromine. Both phase were 4 weeks each.	COWAT; Sentence Repetition; Ten Words Test; Perceptual Speed; Facial Recognition Test; Judgement of Line Orientation	Response ≥ 50% improvement on HDRS at end-treatment. Multivariate analysis conducted between responders and non-responders.	No significant differences between responders and non-responders in baseline cognitive performance.
(37)	36 single MDE 32 recurrent MDE 5 BD	45.6	HDRS-21: 29.2 MADRS: 33.52	4 weeks of antidepressant treatment, followed up 6 months later to assess relapse.	Test Battery for Attentional Performance; Zahlenverbindungstest; D2 Cancellation Task; Selective Attention Test; SCWT; WMS-R Digit Span Forward & Block Forward; WCST.	Response ≥ 50% improvement on HDRS following 4 weeks of treatment. Remission ≤ 10 on HDRS at discharge.	Non-responders and patients who failed to achieve remission prior to discharge were specifically impaired in divided attention at baseline.
(38)	25 MDD 13 HC	32.5 (11.5)	HDRS-17: 21.3 (4.5) MDRS: 29.4 (4.7)	Naturalistic study. Mixed antidepressant treatment and patients followed up 2–6 months after initial assessment.	Digit Symbol Substitution Test; RAVLT; Paired Associates Learning; Pattern Recognition; Spatial Recognition; Delayed Matching to Sample; COWAT; ‘Exclude Letter' Fluency Test; Spatial Working Memory; Tower of London.	Remission defined as HDRS-17 < 8 at follow-up.	At baseline, significantly less psychomotor dysfunction (Digit Symbol Substitution Test) was evident in those who remitted during the follow up period, than those who did not.
(39)	27 MDE, 2 BD-D, 3 BD-NOS	42.2	HDRS: 21.85	3-month open label SSRI trial	Finger Tapping; Stroop; WAIS Digit Symbol; TMT-A, B & B-A; Continuous Performance Test; Buschke Selective Reminding Test; N-Back; A not B Reasoning Test; Letter & Category Fluency; WCST.	Response = ≥ 50% reduction on HDRS24	Responders were significantly better than non-responders on measures of executive functioning, verbal fluency and working memory. Specifically, non-responders were impaired on A not B, N-Back, Letter Fluency and TMT B-A.
(40)	48 MDD	37.96 (10.63)	HDRS-17: 28.25 (5.69)	All participants were treated with SSRIs or SNRIs and followed up approximately 3-4 months later.	Donders Computerized Simple Reaction Time, WMS-R Digit Span; California Verbal Learning Test; Prospective Memory Test; SCWT, Shortened-WCST; COWAT; Modified Six Elements Test.	Remission = at least 50% improvement on HDRS-17 and no longer meeting syndromal criteria.	S-WCST perseverative errors significantly predicted HDRS at follow-up. S-WCST errors and Prospective Memory categories predicted psychosocial outcome (Social and Occupational Functioning Assessment Scale); worse performance predicted poorer outcomes.
(41)	25 MDD	42.8 (14.2)	HDRS-17: 20.2	Open label, non-randomized design. 8 weeks antidepressant treatment-mixed.	IntegNeuro Battery: Motor Tapping Test, Choice Reaction Time Test, Memory Recall & Recognition Test, Maze Task, Letter Fluency, Spot the Real Word Task, Span of Visual Memory Task, Switching of Attention Test, Time Estimation Task, Sustained Attention Task, Digit Span Task, Word Interference Task.	No criteria-primary outcome change in HDRS following 8 weeks of antidepressant treatment.	Linear regression showed that total memory score was significant predictor in model. Higher pre-treatment memory was associated with greater decrease in depressive symptoms.
(42)	86 MDD 55 HCs	44.12 (12.39)	HDRS-21: 22.31 (6.56)	8-week pharmacotherapy - SSRI.	TMT and SCWT	Response ≥ 50% reduction on HDRS Remission < 7 on HDRS	Lower performance in SCWT and TMT-A at admission associated with highest level of depression at end treatment.
(43)	70 depressed patients (41 MDD, 10 dysthymia and 19 both). 57 HCs	SSRI/dual: *R* = 40.2 (11.9) *NR* = 44.1 (14.4) Bupropion: *R* = 40.3 (13.1) *NR* = 39.2 (14.1) HCs = 35.1 (9.8)	HDRS-17: SSRI/dual: *R* = 15.7 (4.4) *NR* = 16.7 (3.9) Bupropion: *R* = 15.8 (3.2) *NR* = 17.2 (4.1)	8–12 weeks of pharmacotherapy treatment-mixed.	COWAT, WAIS Digit Symbol, 4 Choice Reaction Time and SCWT.	Response - HDRS-17 scores reduced by ≥ 50%.	Non-responders to a SSRI or dual therapy showed poorer word fluency than responders, not seen with Bupropion. Longer choice reaction time was also found in non-responders to a SSRI or dual therapy, but the opposite trend was seen for Bupropion. Using a combined index of fluency and reaction time, equal to or above normal, predicted response to a SSRI or dual therapy. In contrast, less than normal performance predicted response to Bupropion alone.
(44)	19 melancholic depression 50 atypical depression 35 undifferentiated MDD 200 HCs	MDD: Melancholic-35.1 (13.4) Atypical-32.4 (12.8) Undifferentiated-33.6 (11.7) HC-33.5 (10.1)	HDRS-17: Melancholic-28.2 (5.9) Atypical-26.0 (5.5) Undifferentiated-25.1 (6.9)	6-week open label trial. “Semi-naturalistic” as respective psychiatrist could select antidepressant and dosage.	TMT A & B; WAIS Digit Symbol Substitution, Digit Span; modified-WCST; Tower of Hanoi; Animal Naming; Immediate Visual Reproduction.	No criteria - Looked at change in HDRS-17 from baseline to end treatment (6 weeks).	No cognitive predictors of HDRS after 6 weeks of treatment.
(45)	36 MDD	35.89 (11.71)	HDRS: 19.22 (3.46)	10-week open label trial of either Escitalopram or Duloxetine	Parametric Go/No-Go Test	% Change in HDRS	More commission errors on the Go/No-Go task predicted better treatment response.
(46)	49 MDD	74.8 (5.6)	HDRS-21: 22.5 Cornell Scale for Depression in Dementia: 18.6	6-week antidepressant treatment - various	Psychomotor retardation measure of HDRS and Initiation/Perseveration sub-score of MDRS.	Remission = Cornell Scale score of less than 7.	Abnormal I/P scores and psychomotor retardation predicted change in depression scores 6 weeks following treatment. Non-remitters had poorer I/P scores than remitters.
(47)	110 MDD	73.78	MADRS: 24.65	Standardized treatment algorithm-3 months of antidepressant treatment & ECT.	TMT A & B; COWAT; Category Fluency; Benton Visual Retention Test; WAIS-R Digit Span.	Remission = MADRS score of less than 7.	Those who remitted had significantly fewer perseverative errors on COWAT and better performance on Digit Span Forward, than non-remitters.
(48)	100 MDD	69.1 (6.9)	MADRS: 21.8 (8.2)	Assessment of depression and cognitive functioning at study entry and 1 year follow up. Pharmacological treatment with STAGED approach.	TMT A & B; Symbol Digit Modalities Test; WMS-R Logical Memory; Recall of Words from Consortium to Establish a Registry of Alzheimer's disease Word-list.	High response defined as MADRS rating change greater than baseline standard deviation of the sample.	High response individuals (1 year) demonstrated better: baseline Logical Memory delayed recall, Logical Memory % retention, Delayed Word-list recall and higher SDMT scores. Poor baseline performance on tests of verbal memory and processing speed associated with reduced treatment response.
(49)	142 psychotic MDD	71.7 (7.8)	HDRS-17: 30.1 (5.4)	12-week double blind RCT of Olanzapine + Sertraline or Olanzapine + placebo	SCWT and Initiation/Perseveration subscale of MDRS.	No criteria. Conducted a series of linear regressions, with executive function and processing speed as the independent variables and change in HDRS-17 as the outcome variable.	Neither executive functioning nor processing speed predicted change in depression scores.

*BACS, Brief Assessment of Cognition in Schizophrenia; BDI, Beck Depression Inventory; BD-D, bipolar disorder–depressed; BD-NOS, bipolar disorder not otherwise specified; CGI, Clinical Global Impression Scale; COWAT, Controlled Oral Word Association Test; DSM-IV, Diagnostic and Statistical Manual of Mental Disorders; Fourth Edition, ES, effect size; HC, healthy control; HDRS, Hamilton Depression Rating Scale; MADRS, Montgomery-Asberg Depression Rating Scale; MATRICS, Measurement and Treatment Research to Improve Cognition in Schizophrenia; MCCB, MATRICS Consensus Cognitive Battery; MDD, major depressive disorder; MDE, major depressive episode; MDRS, Mattis Dementia Rating Scale; MMSE, Mini Mental State Examination; NR, non-responder; QIDS, Quick Inventory of Depressive Symptomatology; R, responder; RAVLT, Rey Auditory-Verbal Learning Test; RVIP, Rapid Visual Information Processing; SCWT, Stroop Color and Word Test; SNRI, Serotonin Noradrenaline Reuptake Inhibitor; SSRI, Selective Serotonin Reuptake Inhibitor; STAGED, Somatic Treatment Algorithm for Geriatric Depression; TMT, Trail Making Test; WAIS-R, Wechsler Adult Intelligence Scale; Revised, WCST, Wisconsin Card Sorting Test; WMS-R, Wechsler Memory Scale-Revised*.

**Table 2:** Reviewed studies using other treatments during the follow-up period.

**Study**	**N**	**Age (SD)**	**Depression severity**	**Study design**	**Cognitive tests**	**Response/remission criteria**	**Key outcomes**
(50)	19 MDD	37.2	HDRS: 26	3-week, randomized trial of CBT or CBT + Sleep deprivation therapy.	D2 Letter Cancellation Test, Test of Attentional Performance, Zahlen Verbindings Test, subtest 6 of German Intelligence Battery, German version of Auditory Verbal Learning Test.	No criteria-primary outcome post treatment HDRS.	For the CBT only group, declarative verbal memory and word fluency predicted clinical response (percentage improvement on HDRS).
(51)	57 MDE	46.7 (11.6)	MADRS: 29.4 (5.4)	Data pooled from 5 clinical trials of transcranial direct current stimulation-2 double-blind (10 and 15 sessions) and 3 open-label (20 sessions).	RAVLT; Digit Span, COWAT; Symbol Digit Modalities Test; Simple & Choice Reaction Time.	No criteria-primary outcome post-treatment MADRS.	Better pre-treatment performance on COWAT associated with better antidepressant response to transcranial direct current stimulation.
(25)	Details in Table 1					
(52)	20 TRD	47.4	HDRS-17: 24.3	12 months of subcallosal cingulate gyrus deep brain stimulation.	WCST, Hopkins Verbal Learning Test, COWAT, Finger Tap Test, Stroop.	Response-HDRS scores reduced by ≥ 50.	Dominant-hand finger tap test and WCST-Total errors predicted treatment response with a high degree of accuracy. Responders performed significantly better on the finger tapping test, but had significantly more errors on the WCST.
(49)	25 MDD	70.80 (5.52)	HDRS-17: 30.19 (5.76)	12-week antidepressant treatment regimen within a 12-month follow-up period. ECT was the final treatment option, with 48% receiving ECT treatment.	WAIS Block Design, Digit Span (forward and backward), Digit Symbol subtests; WMS Logical Memory and Visual Memory subtests; TMT-A; Tower of London.	Remission was defined as a 17-item HDRS score below 8 between the 6-month and the 12-month visit.	A quantitatively similar performance (whether high, average or low) on verbal learning (Visual Memory associative learning) and planning (Tower of London) appeared to predict remission.
(53)	46 MDD	70.78 (7.3)	HDRS-25: 22.54 (2.7)	12-week randomized trial of problem solving therapy or supportive therapy.	Hopkins Verbal Learning Test; WCST; TMT A & B; COWAT, Animal Naming.	Response - HDRS scores reduced by ≥ 50. Remission-post-treatment HDRS score ≤ 10.	Worse performance using empirically derived cut-off score of ≥ 82 on TMT-B, detected 59.6% of psychotherapy treatment responders. Suggests poor baseline switching ability may predict treatment response.
(54)	11 TRD	74.1 (7.81)	MADRS: 25.7 (7.3)	4-week open trial of cognitive remediation (30 h).	TMT A & B; MDRS Initiation/Perseveration subscale; California Verbal Learning Test; WAIS IV Digit Backward.	No criteria - primary outcome change in MADRS.	Higher TMT B - A scores (indicative of greater executive dysfunction) associated with greater reduction in MADRS scores following 4 weeks of cognitive remediation.

*CBT, cognitive behavior therapy; CGI, Clinical Global Impression Scale; COWAT, Controlled Oral Word Association Test; ECT, electroconvulsive therapy; HDRS, Hamilton Depression Rating Scale; MADRS, Montgomery-Asberg Depression Rating Scale; MDD, major depressive disorder; MDE, major depressive episode; MDRS, Mattis Dementia Rating Scale; RAVLT, Rey Auditory-Verbal Learning Test; TMT, Trail Making Test; TRD, treatment resistant depression; WAIS, Wechsler Adult Intelligence Scale; WCST, Wisconsin Card Sorting Test; WMS, Wechsler Memory Scale*.

Studies used a range of cognitive tests and the clinical characteristics of the depressed samples varied substantially across studies. In this review, more emphasis is placed on those studies with the greatest number of participants, as they have more statistical power. While we did not formally rate the quality of studies, we have discussed methodological strengths and weaknesses and taken this into account in synthesizing the evidence. In the sections that follow, the studies will be briefly discussed according to treatment type, and findings will be further divided into different cognitive domains. It is important to note that the way in which tests have been categorized in this review may not align with the cognitive domains described in the original studies; however, it was imperative to organize tests in a standardized way. Given there is considerable overlap in tasks assessing executive function and attention, it was decided to combine both of these cognitive functions together. Further, although working memory is sometimes classified under learning and memory, in this review, it has been classified under the umbrella term of executive functioning. For the purposes of this review, samples containing participants ranging from 18 to 65 years will be referred to as adult samples, and those containing individuals aged 65 and above, will be referred to as older-aged samples.

### Single antidepressant trials

#### Executive function/attention

Eleven studies examined the relationship between executive function/attention and treatment outcomes in 13 adult samples receiving antidepressant monotherapy. Four shorter treatment trials (five samples), examined predictors of response to selective serotonin reuptake inhibitors (SSRIs). Three samples showed an association between poorer executive function/attention performance and poor overall treatment response (total *n* = 268) (13, 14, 20). Conversely, two samples showed no evidence of an association (total *n* = 306) (17, 20). Etkin et al. (20) examined predictors of response to Escitalopram (*n* = 217) and Sertraline (*n* = 234). They found that impairment in attention and working memory was associated with non-remission on Escitalopram; however, this relationship was not seen with Sertraline (20). Of the negative studies, Gudayol- Ferré et al. found no evidence of an association between executive function or attention and overall response to Fluoxetine (17); however, they did find an association with early treatment response and time to remission (16, 18). In their sample of 72 depressed patients, poorer attention and spatial working memory were associated with poorer response at 4 weeks, but the opposite relationship was seen with “subsequent thinking time” on the Stockings of Cambridge (16). Additionally, the authors also found that those with poorer spatial working memory performance were slower to remit at treatment-end; thus, their findings with respect to attention and spatial working memory are in line with the other positive studies (18).

In a longer treatment trial, Bastos et al. (25) examined the relationship between executive function performance and response to 24 months of treatment with Fluoxetine (*n* = 91), psychodynamic psychotherapy (*n* = 90) or a combination thereof (*n* = 90). The largely negative results were complex. Of 14 cognitive variables, higher scores on two (WAIS-III, Letter Number Sequencing and Matrix Reasoning) were associated with better response across all three treatments (Fluoxetine, psychodynamic psychotherapy and the combination) and higher scores on one (WAIS-III, Similarities) was associated with poorer response (25).

Whilst one small study has found an association between poorer executive function/attention performance and poor response to SNRIs (*n* = 25) (21), a much larger study has found no evidence of a relationship (*n* = 204) (20).

In a naturalistic multi-center trial of 6–8 weeks of Agomelatine (an antidepressant with a primarily melatonergic action) treatment (*n* = 508), the number of omissions on the D2 Cancellation Task (a measure of attention) predicted clinical and functional remission in patients with moderate to severe depression. Moreover, a dose-response relationship was observed, whereby treatment outcomes were increasingly more positive as less omission errors were made on the task (24).

One study has examined the relationship between baseline executive measures and response to the combined dopamine and noradrenaline re-uptake inhibitor, Bupropion. Herrera-Guzmán et al. (*n* = 26) found that poorer performance on the Stockings of Cambridge at baseline predicted poorer response to 8 weeks of Bupropion treatment (15).

Three studies have examined cognitive predictors of Ketamine response, with two (total *n* = 38) finding evidence of an association between poorer executive function/attention performance and better treatment response (19, 22). Murrough et al. (22) (*n* = 25) found that responders to a single infusion of Ketamine Hydrochloride performed significantly worse on tests assessing working memory, than non-responders (22). In line with this finding, Shiroma et al. (19) found that the likelihood of responding to six infusions of Ketamine was greater in those who demonstrated poorer attentional abilities at baseline (19). In contrast, a second study by Murrough et al. (*n* = 43), showed no association between executive function/attention performance and treatment response (23).

Seven studies examined the relationship between executive function/attention and treatment-related outcomes in response to antidepressant (SSRI) monotherapy in older-aged samples. Five of the studies found that deficits in executive functioning were associated with poor remission rates/antidepressant response (combined *n* = 341) (26, 28, 29, 32, 34, 35). In contrast, one small study (*n* = 12) found no difference in executive function performance between remitters and non-remitters (30). One study (*n* = 13) examined the relationship between executive function/attention and time to remission and found that individuals with impaired executive functioning, as shown by greater conflict scores on the Attention Network Test, took longer to remit (31).

#### Psychomotor speed

Ten studies examined the relationship between psychomotor speed and response to antidepressant monotherapy, in 12 adult samples. Four shorter treatment trials examined predictors of response to SSRIs. In two samples (14, 20), slower psychomotor speed was associated with poorer response to treatment (total *n* = 254). In contrast, three samples showed no association between SSRI treatment and psychomotor speed (*n* = 320) (13, 17, 20). In the large study by Etkin et al. slower psychomotor speed was associated with non-remission to Escitalopram, but not to Sertraline (20). One study examined the relationship between psychomotor speed and 24 months of treatment with an SSRI (25). The study found that slower psychomotor speed was associated with poorer response to Fluoxetine treatment (*n* = 91).

No association was found between psychomotor speed and response to SNRIs (*n* = 204) (20), Bupropion (*n* = 26) (15) and Agomelatine (*n* = 508) (24). Two studies (total *n* = 68) (22, 23) examining the relationship between psychomotor speed and response to Ketamine found that slower psychomotor speed at baseline predicted greater improvement in depressive symptoms following treatment. Conversely, one Ketamine study (*n* = 13) found no association (19).

Three studies have examined the relationship between psychomotor speed and overall treatment response in older-aged adults. None of the studies (total *n* = 594) found an association between psychomotor speed and treatment response (27, 33, 34). However, one study (*n* = 84) did find that slower psychomotor speed was associated with slower response to treatment. Sneed et al. (33) found that individuals with slower psychomotor speed took longer to respond to Citalopram than those with faster psychomotor speed; however, by the end of treatment (week 8), both groups were equal in their level of response (33).

#### Verbal learning and memory

Eight studies examined the relationship between verbal learning and memory, and treatment-related outcomes in response to antidepressant monotherapy in adult samples. Seven shorter treatment trials (total *n* = 885) (13, 15, 17, 19, 22, 23), plus one long-term treatment trial (*n* = 91) (25), found no evidence of an association between the two. Likewise, the four studies that examined verbal learning and memory in older-aged samples (total *n* = 616) found no relationship between verbal learning and memory performance and treatment-related outcomes (26, 27, 33, 34).

#### Non-verbal learning and memory

Seven studies examined the relationship between non-verbal learning and memory performance and treatment-related outcomes in response to antidepressant monotherapy in adult samples. Six shorter studies (total *n* = 204) (13, 14, 17, 19, 22, 23) and one longer-term study (*n* = 91) (25) found no relationship between non-verbal learning and memory, and treatment response. One study (*n* = 26) found that poorer non-verbal memory performance was associated with better response to a combined dopamine and noradrenaline re-uptake inhibitor (26). In their 8-week trial of Bupropion, Herrera-Guzmán et al. found that responders performed significantly worse on a measure of visual memory (Paired Associates Learning) than non-responders (15). One study has examined the association between non-verbal learning and treatment response in older-aged depression (*n* = 22); however, they found no evidence of a relationship between the two (26).

#### Emotional processing

Only two studies have examined the predictive nature of emotional processing in relation to treatment response in depression. In a younger adult sample, Etkin et al. (20) found that slower emotion identification speed was associated with non-remission to Escitalopram (*n* = 217), but not Sertraline (*n* = 234) or Venlafaxine (*n* = 204) (20). In an older-aged sample (*n* = 12), Alexopoulos et al. (30) found no differences between remitters and non-remitters in terms of their performance on an emotional go/no-go task following 8 weeks of treatment with Escitalopram (30).

### Mixed antidepressant treatment

#### Executive function/attention

Ten studies examined the relationship between executive function/attention and response to mixed antidepressant treatment in 11 adult samples with depression. In five of the samples (total *n* = 291), poorer executive function/attention was associated with poorer response to various pharmacological treatments (37, 39, 40, 42, 43). In a sample of particularly severely depressed inpatients, Whithall et al. (40) found that poorer executive function performance was associated with negative clinical and functional outcomes in inpatients treated with SSRIs or SNRIs. More perseverative errors on the shortened Wisconsin Card Sorting Test (WCST) at baseline, was associated with greater depression severity at follow-up. Further, more perseverative errors on the WCST in addition to poorer event-based prospective memory, was associated with poorer social and occupational outcomes in their sample (40). In contrast, one small study (*n* = 36) found the opposite relationship between executive function performance and treatment response. Crane et al. (45) found that more commission errors on the Parametric Go/No-Go test predicted better treatment response to Escitalopram or Duloxetine (45). Four samples (total *n* = 228) showed no association between executive function/attention performance and overall treatment response (36, 38, 41, 43, 44).

Four studies examined the relationship between executive function/attention and treatment response in older-aged samples. Two studies (total *n* = 159) found an association between executive dysfunction and poor treatment response (46, 47). In the largest positive study, Potter et al. (47) (*n* = 110) found that remitters to a standardized treatment algorithm over 3 months had significantly fewer perseverative errors on the Controlled Oral Word Association Task (COWAT) and better performance on Digit Span Forward, than non-remitters (47). Story et al. (48) (*n* = 177) examined response to a standardized treatment algorithm over one year and found no association with executive function performance (48). Additionally, in a 12-week randomized controlled trial (RCT) comparing Olanzapine plus Sertraline with Olanzapine plus placebo (*n* = 142), Bingham et al. (49) found no association between baseline executive functioning and depression scores post treatment (49). This study did not present data separately for the two treatment groups.

#### Psychomotor speed

Eight studies examined the relationship between psychomotor speed and treatment outcomes with mixed antidepressant treatment in nine adult samples. Three samples (total *n* = 159) showed an association between slower psychomotor speed and poorer response to treatment (38, 42, 43). In the largest positive study (*n* = 86), slower performance on Part A of the Trail Making Test (TMT) was associated with greater depressive symptomatology following 8 weeks of SSRI treatment in a sample of adults with severe depression (42). In contrast, six samples (total *n* = 283) showed no association between psychomotor speed and treatment response (39, 41, 43, 44). In a sample of 104 individuals with depression, Lin et al. (44) found no association between psychomotor speed and improvement in HDRS scores following 6 weeks of treatment (44).

Four studies examined the relationship between psychomotor speed and treatment response in older-aged samples. One 6-week study (*n* = 49) and one 12-month study (*n* = 177) found an association between poorer psychomotor speed and poor treatment response (46, 48), whilst two studies (*n* = 252) did not (47). In the larger positive study (*n* = 177), Story et al. (48) found that depressed older persons with better baseline performance on the Symbol Digit Modalities Test, showed the greatest improvement in depressive symptomatology at one-year follow-up (48).

#### Verbal learning and memory

Five studies have examined the predictive value of verbal learning and memory in relation to mixed antidepressant treatment in adult samples. Spronk et al. (41) found that higher pre-treatment verbal memory performance was associated with a greater reduction in depressive symptoms, in a sample of 25 individuals with major depressive disorder (MDD) (41). Four other studies found no association between verbal memory and treatment response (total *n* = 157) (38–40).

Two studies have examined the relationship between verbal learning and memory, and treatment response in older-aged adults. Story et al. (48) (*n* = 177) found that depressed older adults who performed well on verbal memory tasks prior to being treated with a stepped approach, showed the greatest improvement in depressive symptomatology at 1-year follow-up (48). However, another study (*n* = 110) found no relationship at 3-month follow-up (47).

#### Non-verbal learning and memory

Three studies examined the relationship between non-verbal learning and memory and treatment outcomes with mixed antidepressant treatment in adults (38, 44), and none of the studies found evidence that non-verbal learning and memory predicts treatment response (total *n* = 181). Likewise, the single study that examined non-verbal learning and memory in older-aged samples found no association between the two (*n* = 110) (47).

#### Emotional processing

Only one study has examined the relationship between emotional processing and treatment outcomes with mixed antidepressant treatment in adults with depression. In the study by de Groot et al. (36), there was no significant difference between responders and non-responders in terms of their performance on a facial expression recognition task at baseline (36).

### Other biological treatments

Two studies have examined cognitive predictors (executive function, verbal learning and memory, and psychomotor speed) of treatment response to other biological treatments in younger adult populations. Martin et al. (51) pooled data (total sample, *n* = 57) from five clinical trials of anodal transcranial direct current stimulation and found that better baseline performance on the COWAT (a measure of executive functioning), was associated with better response to transcranial stimulation (51). McInerney et al. (52) examined cognitive predictors of 12 months of subcallosal cingulate gyrus deep brain stimulation (*n* = 20) and found that better psychomotor speed, but greater executive dysfunction, predicted better response to treatment (52).

One study examined cognitive predictors of treatment response to other biological treatments in older-aged individuals. Marcos et al. (55) assessed the predictive value of executive functioning/attention, verbal learning and memory, non-verbal learning and memory, and psychomotor speed, in a 12-week antidepressant trial (*n* = 25). ECT was available if patients failed to respond to pharmacological treatment, with 48% of the sample receiving ECT at some point during the trial. The authors found no association between cognitive function and response to treatment (55).

### Psychosocial treatments

Two studies have examined cognitive predictors of psychosocial treatment response in adults with depression (25, 50). Kundermann (50) examined the predictive value of executive function/attention, verbal learning and memory and psychomotor speed, in a 3-week trial of Cognitive Behavior Therapy (CBT) vs. CBT + sleep deprivation therapy (*n* = 19). The authors found that better verbal fluency and declarative verbal memory were associated with better clinical response (percentage improvement on the HDRS) in those receiving CBT alone. However, this relationship was not seen in the group receiving CBT + sleep deprivation therapy. No association was found between psychomotor speed and treatment response in either of the two groups (50).

Bastos et al. (25) examined the relationship between executive function, verbal learning and memory, and processing speed, and response to 24 months of psychodynamic therapy (*n* = 90), Fluoxetine (*n* = 91) or psychodynamic therapy with adjunctive Fluoxetine treatment (*n* = 90). The authors found mixed findings in relation to the predictive value of executive functioning. Across all three treatment groups, better Letter-Number Sequencing and Matrix Reasoning scores, predicted lower depression symptoms (BDI) at 24 months. Conversely, better Similarities scores were associated with greater depression symptoms following treatment. The authors also found that in those receiving psychodynamic therapy or both treatments combined, better Digit-Symbol Coding scores (i.e., faster psychomotor speed) were associated with greater depression severity at follow-up (25).

Two studies have examined the association between cognitive function and response to psychotherapy in older-aged samples with depression (53, 54). Both examined executive functioning/attention, verbal learning and memory, and psychomotor speed. Both studies found executive functioning to be the only domain associated with treatment-related outcomes. Beaudreau et al. (53) (*n* = 46) found that poor baseline performance on Part B of the TMT (a measure of cognitive flexibility), detected 59.6% of individuals who responded to 12 weeks of either problem-solving therapy or supportive therapy. In a 4-week trial of cognitive remediation, Morimoto et al. (54) found that higher TMT B-TMT A scores (indicative of greater executive dysfunction) was associated with greater reduction in Montgomery-Asberg Depression Rating Scale (MADRS) scores following treatment (54).

## Discussion

### Summary of results

Since different treatments may act differently on brain circuitry and there is evidence that modulation of specific receptors or circuits may differentially affect cognitive function, it is important in the first instance to divide the results of the review into studies examining response to different treatment modalities. In addition, whilst this review excluded studies that included patients with likely onset of dementia (MMSE <24), changes associated with aging, multiple episodes of depression or late onset depression may result in a different pattern of association in older samples. Therefore, we have separated the results into adult and older-aged samples. In summary, the results are as follows:

#### Executive function/attention

There was some consistency in findings from studies examining response to SSRIs in adult samples. Three samples treated with a single SSRI (13, 14, 20) (total *n* = 268) and two samples openly treated with any SSRI (*n* = 123) (39, 42) showed that reduced executive function was associated with poorer response. In contrast, two single SSRI studies (total *n* = 306) showed no association (17, 20). In older-aged samples, the findings were much more consistent. Five studies showed an association between executive function and overall response to SSRIs (total *n* = 341) (26, 28, 29, 32, 34, 35), and one found an association between executive function performance and time to remission (31). Only one small study (*n* = 12) found no differences in executive functioning between remitters and non-remitters (30).

In terms of other agents, one large study found that poorer attention was associated with poorer response to a melatonergic agent in an adult sample (*n* = 508) (24). There was limited, or no evidence, of an association between executive function/attention and response to SNRIs, Bupropion, Ketamine, ECT or psychosocial treatments.

#### Psychomotor speed

There was some evidence of a relationship between psychomotor function and response to SSRIs in adult samples. Two samples (total *n* = 254) showed that slower psychomotor speed was associated with poorer response (14, 20). Additionally, one longer-term SSRI study (*n* = 91) (25) and one sample openly treated with any SSRI (*n* = 86) (42), showed the same association. In contrast, three adult samples (*n* = 320) treated with a single SSRI (13, 17, 20) and one sample openly treated with any SSRI (39), showed no association. There was no association in older-aged adults.

There was limited, or no evidence, of an association between psychomotor speed and response to SNRIs, Bupropion, Ketamine, Agomelatine, ECT or psychosocial treatments in adult or older-aged samples.

#### Learning and memory

There was limited evidence of a relationship between learning and memory (verbal or non-verbal) and treatment response in adult or older-aged samples.

### Prediction of response to monoamine reuptake inhibitors

As noted in the summary above, the data in younger participants is dominated by the large study of Etkin et al. (20). The sample randomly assigned to Escitalopram showed that executive dysfunction was associated with poorer response, while there was no such association for the other SSRI, Sertraline (20). The authors speculated that the difference between Escitalopram and Sertraline may relate to the exact pharmacodynamic properties of the agents, the suggestion being that Escitalopram is a more specific SSRI while Sertraline has more noradrenergic and dopaminergic reuptake inhibition. The other related possibility is that their findings were related to dose; however, the validity of such explanations is not clear. The doses of all three antidepressants in the international Study to Predict Optimized Treatment in Depression (iSPOT) study were low (Escitalopram 12 mg, Sertraline 62 mg, Venlafaxine 83 mg) (56). Evidence does not suggest that Venlafaxine has significant effects on noradrenaline re-uptake at this dose (57, 58). *In vitro*, Sertraline has been shown to inhibit noradrenaline and dopamine reuptake (59), but the extent to which this occurs at the doses used in this study *in vivo* is unclear. Furthermore, neither of these factors can explain the differential response whereby those with poorer cognitive function were more likely to remit with Sertraline/Venlafaxine and those with better cognitive function were more likely to remit with Escitalopram. In the iSPOT study, and in meta-analyses, there was no difference in overall efficacy between these three antidepressants (56, 60). The analysis used in this study was different from that used in all other studies, using a cross-validated multivariate pattern classification approach. This allows different variables to be weighted differentially in order to obtain the best predictive model. As such, it is significantly different from the simpler methods of examining association and less comparable than the results from other studies.

Results for processing speed are similar to those for executive function, with the largest study showing a relationship between processing speed and response to treatment with Escitalopram but not Sertraline or Venlafaxine (20). It has been suggested that reduced psychomotor function indicates a particular subtype of depression; melancholic depression (61). Further, it is suggested that this responds preferentially to dual action drugs compared with SSRIs or psychotherapy (62, 63). The association with response to Escitalopram but not Venlafaxine could therefore relate to a poor response of “melancholic” patients—in this context indicated by psychomotor impairment—to Escitalopram but not Venlafaxine. However, as noted, Venlafaxine at this dose may vary little from a standard SSRI. It has been suggested that measuring psychomotor function either by testing or observation, is a better way of assessing a measurably different (“melancholic”) group.

### Response to other agents and biological treatments

A large study examining predictors of response to Agomelatine (an antidepressant with a primarily melatonergic action), showed that those who performed better on an attentional task were more likely to achieve both clinical and functional remission (24). The authors postulated that the ability to direct attentional resources toward, and away from, emotionally-laden stimuli, is critical for effective emotional regulation. Thus, attentional deficits would likely impact one's ability to regulate emotion; thereby, contributing to persistent negative affect (24).

Interestingly, studies examining Ketamine found the opposite relationship between cognitive functioning and treatment response, with poorer neurocognitive performance, particularly slower psychomotor speed, predicting greater improvement in depressive symptoms (19, 22, 23). While preliminary, the findings suggest that responders to Ketamine may show a distinct cognitive profile compared with those who respond to other types of antidepressants, such as SSRIs. Dopaminergic transmission within prefrontal-subcortical circuits has been implicated in several cognitive processes, including psychomotor function (64); further, Ketamine has been shown to modulate dopamine transmission within these brain regions (65, 66). Although Ketamine's exact mechanism of action is yet to be fully elucidated, it is possible that its antidepressant effects are through modulation of dopaminergic signaling. In line with this, Bupropion is a relatively specific dopamine and noradrenaline reuptake inhibitor, and Herrera-Guzmán et al. (15) found that poorer executive function performance predicted better response to Bupropion (*n* = 26).

### Response to psychotherapy

Few studies have examined cognitive predictors of response to psychotherapy. Focusing on the short-term studies, one study found that better executive functioning and verbal memory were associated with better response to treatment with CBT (50). Conversely, two studies found that executive dysfunction predicted better response to Problem Solving Therapy (PST) and supportive therapy (67), and cognitive remediation (54). The discrepancy in findings may be due to the nature of the psychosocial treatments being used. The latter studies incorporated treatments that either targeted executive dysfunction and its underlying pathophysiology (PST and cognitive remediation) or did not rely heavily on executive processes (supportive therapy). Therefore, it is possible that treatments which support or improve executive functioning, may facilitate clinical improvement in those experiencing such cognitive deficits.

### Data in older-age samples

Particularly for executive function, data in older-age samples are remarkably consistent. Most studies showed that impaired executive function was associated with poorer response to treatment. It is possible that this may be related to the number of previous episodes, as both cognitive functioning and treatment response decline with increasing depressive episodes (68, 69). Some authors have suggested that executive dysfunction is particularly prominent in the older-age individuals (70), although not all studies have agreed (71). If executive function is particularly prominent or frequent in these samples, then this would reduce the likely dilution effect of including patients with minimal deficit. One large study which illustrates this effect was excluded from this review based on their use of a different measure of response (functional measure) (12). A treatment specifically designed to counteract the negative prognostic effect of executive deficit, Problem Solving Therapy (PST), was compared with supportive therapy in a sample of old-age depressed patients. An advantage was seen for PST, particularly in those patients with greater executive deficit. This study is unique because it is enriched specifically for executive impairment. It therefore addresses an issue which is particularly important in this area—the dilution of findings by inclusion of patients without cognitive impairment (72).

### Neurobiological underpinnings

As mentioned above, there appears to be some support for the notion that executive dysfunction can predict treatment-related outcomes, particularly in the elderly depressed; with deficits in executive functioning/attention predicting poorer or slower response to treatment. One reason for this finding may be that impaired executive function performance serves as a marker for dysfunction within the fronto-limbic circuits. Executive functioning is sub-served by areas within the prefrontal cortex and there is consistent evidence that depression is associated with aberrant neural activity in these brain regions (73, 74). It has been proposed that reduced prefrontal control over limbic activity leads to impaired emotional regulation and maladaptive thinking patterns, such as rumination and worry, all of which are believed to contribute to the development and maintenance of depression (75, 76). Thus, poor performance on executive function tasks may highlight key pathological processes that serve to not only maintain depression but preclude response to treatment.

### Methodological issues

Although not an exhaustive list, the following section will discuss the most pertinent methodological considerations related to the studies included in the current review.

1. Standardized monotherapy vs. open label trials and algorithm-based treatment—We have dealt with the results based on the treatment used. It is important to note that there is a fundamental difference between a trial of a single agent and one which allows changes in treatment based on tolerance and response. In monotherapy, for example, patients may not respond because they cannot tolerate the treatment; something which is unlikely to be directly related to cognitive function. In contrast, open and algorithm-based treatment trials permit changes to treatment if an agent is not tolerated, and the patient may still be classified as a responder. These trials have greater ecological validity and may more accurately reflect the clinical implications of cognitive impairment in determining real-life response. However, they do not give accurate information regarding likelihood of response to one agent compared with another.
2. Length of trial–There is a marked difference between the timing of the clinical outcome between trials (in this review ranging from 24 h to 1 year). On one hand, a short outcome may constrain the time available to respond, making differentiation between patients less likely. However, longer outcome times (e.g., 1 year) allow various other factors to operate, thereby reducing the likelihood of finding a clear association. For example, patients in such studies may respond and then relapse within the time frame of the study. In older-age samples, a longer outcome may also involve development or progression of a neurodegenerative process, meaning that cognitive impairment is in fact associated with further cognitive decline.
3. Choice of outcome measure–Trials have used a variety of ways of measuring response, including percentage reduction in mood rating scale scores, rates of response and rates of remission. The former keeps response on a dimension and is likely to be more sensitive than a binary outcome. Especially in short trials, remission is relatively infrequent and reduces the sensitivity of the analysis; however, it is the optimal and arguably most clinically-relevant outcome. Another related issue is the utilization of multiple outcome measures. This increases the number of comparisons being made and the likelihood that an association may be purely due to chance.
4. Severity of depression at baseline–There are several issues regarding depression severity at baseline. Firstly, placebo response tends to be greater in milder depression, possibly indicating less of a biological basis (77). Response in this case is less likely to be biologically determined and therefore, less likely to be influenced by cognitive impairment. Secondly, a related issue is that in mild to moderate depression, the percentage of patients who have cognitive impairment is relatively low (78); hence, using such a sample will likely dilute findings (see below). Thirdly, in milder depression, the range of possible change on depression rating scales is smaller, which ultimately reduces the likelihood of finding an association between baseline cognitive functioning and change in depressive symptomatology.
5. A related issue is the way in which severity of depression has been accounted for in the analysis of the relationship between cognitive variables and outcome. This is clearly important since the relationship between cognitive function and outcome may be mediated wholly or partly by severity of depression. Most large studies accounted for this by some form of covariate analysis. Other studies simply compared the baseline depression rating score of responders compared with non-responders, and if this was not significantly different, concluded that this was not an important mediator of difference in cognitive function. Clearly, this issue should be addressed in future research.
6. Degree of cognitive impairment at baseline–It is less likely that cognitive function will be associated with outcome in patients whom are not classed as “cognitively impaired.” Having such patients in a study will dilute findings, potentially to the point of a genuine association not being demonstrated. Some of the reviewed studies had control participants and indeed found a difference between controls and the group overall (20, 79), but this does not mean that all, or even a high percentage of patients, were impaired (78). It is likely that studies including more severely unwell patients have a higher percentage of patients with significant cognitive impairment and are therefore, more likely to show an association with response. In the study of Etkin et al. (20), the importance of this phenomenon is illustrated, with the predictive effect of cognitive performance only applied in the group who, compared with healthy controls, were significantly impaired.
7. Cognitive battery–Studies using a more extensive battery of cognitive tasks may be more likely to show an association with response simply because they have used multiple tests, and an association with one of these may simply be a feature of multiple comparisons. However, it could be argued that certain aspects of, for example executive function, may be more likely to affect response than others. Pimontel et al. (80), in a meta-analysis of executive function tasks in the elderly, conclude that only planning and organization (as measured by a subtest of the Dementia Rating Scale) was associated with response. Using composite scores for each domain may reduce the problem of multiple comparisons, but as noted, some may argue that this neglects individual aspects of cognitive functioning. Very short batteries may measure cognitive domains inadequately and result in false negative findings. Additionally, cognitive tasks themselves may vary in their sensitivity and suffer from ceiling effects. For example, the Hopkins Verbal Learning Test may not measure verbal learning and memory with adequate sensitivity, and even the Rey Auditory Verbal Learning Test may be subject to ceiling effects; thus, more sensitive tasks are needed (81).
8. Classification of drop-outs–Outcome can be analyzed either by intention-to-treat or using only completers. Most studies in the review classified patients as responders or remitters based on pre-defined criteria and classified drop-outs as non-responders/remitters. A small number, including one of the largest studies in the review (20), examined change in depression rating scale scores and therefore included in the analysis only patients who completed follow-up rating scales. However, they also undertook an intention-to-treat analysis and a sensitivity analysis, showing that the method of analysis made no difference in this case. The advantage of the former is that it includes all patients and gives a potentially more useful clinical result informing what the likely overall outcome is for patients with differing cognitive profiles. However, the relationship between cognitive function and outcome may be altered by the group of patients who are particularly sensitive to side effects which may not relate to cognitive function.

### Limitations

There are several limitations of the current review. Firstly, as is usual in English language-based reviews, only peer-reviewed articles in the English language were included, which may have resulted in some useful sources of evidence being missed. Secondly, given the heterogeneity of outcome measures, the variety of treatment protocols, and the differing ways in which data was presented and analyzed, it was not possible to use a meta-analytic technique. This meant that a quantitative result could not be produced. Thirdly, no formal risk of bias methodology was utilized in the present review (e.g., Cochrane risk of bias tool). However, differences in assessment were discussed between the three co-authors and studies of greater quality were given greater weight in synthesizing the evidence. Further, methodological issues related to the reviewed studies are discussed.

### Recommendations for future research

1. Studies examining this issue will have low yield unless they have a significant proportion of patients with significant cognitive impairment. Selection of patients may require a strategy to enrich samples, or simply to recruit more severely depressed samples.
2. The issue of chance findings is important. We suggest that studies employ *a priori* groupings of variables into domains and utilize composite domain scores in analysis. This reduces the number of variables examined. Secondary analyses can examine individual variables to elicit any more specific signals regarding detailed cognitive functions which may be associated with response.
3. The majority of studies have employed outcome measures of response or remission. Although it can be argued that these are more clinically meaningful, examining response on a dimensional scale (i.e., percentage change in mood rating scale scores) is likely to be more sensitive; thereby, increasing the likelihood of detecting an association between cognitive functioning and response. One way to deal with this issue is to be clear regarding which primary outcome is to be related to cognitive function but to report other associations in secondary analyses, thereby making these data easily accessible for meta-analyses, but avoiding the problem of multiple outcomes analyses.
4. It appears that “cold” (i.e., traditional) cognitive functions have been the main focus in this area of research, with only three studies having assessed the predictive nature of emotional processing. There is strong evidence that depressed individuals experience alterations in emotional processing (e.g., negatively interpreting emotionally laden stimuli) (76, 82). Moreover, “hot” (i.e., emotional) cognitive processes are believed to play a role in the development and maintenance of depressive symptoms. Thus, more focus should be placed on assessing the relationship between emotional processing and treatment response.

### Summary, conclusions and clinical implications

In younger patients, the data is inconclusive both regarding the association between cognitive function and response to any treatment, and regarding association with response to specific treatments. The best evidence is for a predictive effect of executive function, and with some support for an association with psychomotor function. There is no evidence of learning or memory being associated with treatment response. The main methodological issue we believe is that samples were relatively mildly depressed and therefore, likely contained few patients with significant cognitive impairment. The evidence in older adults is much more consistent and suggests that poor executive function predicts poor response to SSRIs, with little evidence regarding response to other agents. In line with this, one notable study showed that specifically addressing executive dysfunction in the elderly depressed, had positive effects (12).

It is apparent that this area of research is affected by a number of important methodological issues, which need to be addressed in order to help fully elucidate the relationship between cognitive functioning and treatment outcomes in depression. Nevertheless, the findings from the present review do suggest that certain aspects of cognitive functioning, particularly executive function, may be useful in predicting treatment response in depression. This is certainly the case in older-aged samples, with evidence suggesting that executive dysfunction can predict poor response to SSRI treatment. The findings also indicate a possible rationale for specifically targeting cognitive functioning during treatment, as doing so may result in improved treatment outcomes.

## Author contributions

SG conducted the systematic review of papers and prepared the first draft of the manuscript. RP and KD supervised the systematic review and reviewed and updated subsequent drafts.

### Conflict of interest statement

The authors declare that the research was conducted in the absence of any commercial or financial relationships that could be construed as a potential conflict of interest. The reviewer RS and handling Editor declared their shared affiliation at the time of the review.
